# Supplementary material for: Dual Targeting Factors Are Required for LXG Toxin Export by the Bacterial Type VIIb Secretion System
Source: mBio. 2022 Aug 29;13(5):e02137-22. doi: 10.1128/mbio.02137-22 (PMC9600955; doi:10.1128/mbio.02137-22)
Supplement: FIG S1 [file mbio.02137-22-s0001.pdf]

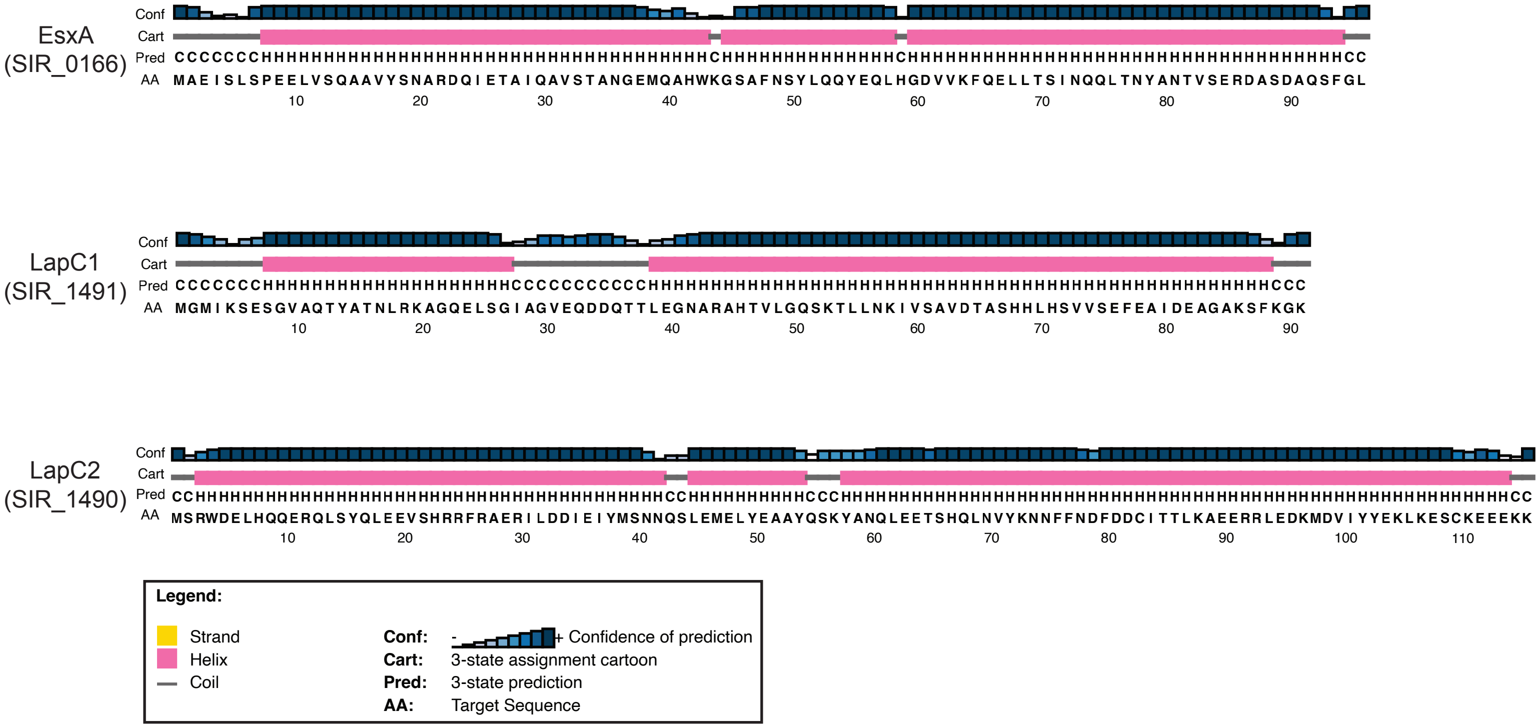
EsxA (SIR_0166)
Conf
Cart
Pred CCCCCCCHHHHHHHHHHHHHHHHHHHHHHHHHHHHHHHHHHHHCHHHHHHHHHHHHHHCHHHHHHHHHHHHHHHHHHHHHHHHHHHHHHHHHHHCC
AA MAEISLSPEELVSQAAVYSNARDQIETAIQAVSTANGEMQAHWKGSAFNSYLQQYEQLHGDVVKFQELLTSINQQLTNYANTVSERDASDAQSFGL
LapC1 (SIR_1491)
Conf
Cart
Pred CCCCCCCHHHHHHHHHHHHHHHHHHHHCCCCCCCCCCCHHHHHHHHHHHHHHHHHHHHHHHHHHHHHHHHHHHHHHHHHHHHHHHHHHHHHHCCC
AA MGMIKSESGVAQTYATNLRKAGGQELSGIAGVEQDDQTTLEGNARAHTVLGQSKTLLNKIVSAVDTASHHLHSVVSEFEAIDEAGAKSFKGK
LapC2 (SIR_1490)
Conf
Cart
Pred CCHHHHHHHHHHHHHHHHHHHHHHHHHHHHHHHHHHHHHHHHCCHHHHHHHHHHCCCHHHHHHHHHHHHHHHHHHHHHHHHHHHHHHHHHHHHHHHHHHHHHHHHHHHHHHHHHHCC
AA MSRWDELHQQERQLSYQLEEVSHRRFRAERILDDIEIYMSNNQSLEMELYEAAYQSKYANQLEETSHQLNVYKNNFFNDFDDCITTLKAEERRLEDKMDVIYYEKLKESCKEEEKK
Legend:
Strand
Helix
Coil
Conf: - + Confidence of prediction
Cart: 3-state assignment cartoon
Pred: 3-state prediction
AA: Target Sequence
