# Supplementary material for: Dual Targeting Factors Are Required for LXG Toxin Export by the Bacterial Type VIIb Secretion System
Source: mBio. 2022 Aug 29;13(5):e02137-22. doi: 10.1128/mbio.02137-22 (PMC9600955; doi:10.1128/mbio.02137-22)
Supplement: TABLE S1 [file mbio.02137-22-s0007.pdf]

Table S1. Spectral counts for TelC-V and LapC1-V immunoprecipitated samples and their respective control samples.

#	Identified Proteins	Accession Number	Alternate ID	Δ*telC* ctrl	Δ*telC* + *telC*-V	Δ*wxgC* ctrl	Δ*wxgC*+ *wxgC*-V
1	TelC-VSV-G tagged	TelC_VSV-G		0	716	11	191
2	WxgC-VSV-G tagged	WxgC_VSV-G		0	288	2	184
3	Enolase	T1ZFF4	eno	35	45	28	47
4	Polyribonucleotide nucleotidyltransferase	T1ZFD6	pnpA	39	37	35	36
5	Uncharacterized protein	T1ZGI6	SIR_1490	0	73	0	28
6	Oligopeptide-binding protein AmiA	T1ZFZ2	amiA	21	25	12	15
7	60 kDa chaperonin	T1ZGB6	groL	20	20	8	15
8	ABC-type transport system, periplasmic binding protein	T1ZEB9	SIR_1223	13	16	9	11
9	Uncharacterized protein	T1ZDT0	SIR_1033	13	10	6	10
10	Putative extracellular solute-binding protein	T1ZFL7	SIR_1387	11	11	7	11
11	Chaperone protein DnaJ	T1ZG02	dnaJ	10	9	12	13
12	ABC transporter, substrate-binding protein	T1ZG31	SIR_1454	9	13	5	10
13	Isoprenyl transferase	T1ZGQ4	uppS	5	13	7	8
14	Uncharacterized protein	T1ZGR7	SIR_1322	7	13	2	5
15	Uncharacterized protein	T1ZEQ3	SIR_0983	13	7	4	5
16	Foldase protein PrsA	T1ZG93	prsA	8	7	4	7
17	30S ribosomal protein S2	T1ZC88	rpsB	2	15	5	3
18	Aminopeptidase	T1ZEN3	pepC	0	21	0	6
19	Pullulanase, type I	T1ZEI5	pulA	11	5	7	5
20	Elongation factor Tu	T1ZEN1	tuf	4	9	4	5
21	Protein RecA	T1ZFX5	recA	6	15	0	4
22	Ribosomal RNA small subunit methyltransferase H	T1ZGX5	mraW	6	13	5	3

23	Putative rhamnosyltransferase RgpA	T1ZEF3	rgpA	0	9	7	7
24	30S ribosomal protein S5	T1ZGK0	rpsE	4	8	5	7
25	Oxidoreductase	T1ZD40	SIR_0796	5	4	8	2
26	Translation initiation factor IF-3	T1ZDK2	infC	4	8	0	4
27	Uracil phosphoribosyltransferase	T1ZGI0	upp	3	9	2	4
28	Putative lipoprotein	T1ZEA9	SIR_0850	4	6	6	2
29	DNA-directed RNA polymerase subunit beta'	T1ZFL9	rpoC	4	0	5	5
30	Response regulator	T1ZH01	comE	4	11	2	0
31	Beta-N-acetylhexosaminidase	T1ZED9	lacZ	4	5	2	2
32	Beta-N-acetylhexosaminidase	T1ZED9-DECOY		6	0	4	2
33	L-lactate dehydrogenase	T1ZEP5	ldh	3	7	4	4
34	50S ribosomal protein L6	T1ZGX1	rplF	2	9	0	0
35	30S ribosomal protein S12	T1ZCJ3	rpsL	4	3	4	4
36	50S ribosomal protein L4	T1ZFT4	rplD	3	2	2	7
37	DNA-binding protein HU	T1ZCZ7	SIR_0424	6	2	3	6
38	Uncharacterized protein	T1ZGF5	SIR_1455	2	11	0	0
39	Biotin carboxylase	T1ZEV4	accC	3	7	2	3
40	Uncharacterized protein	T1ZBA4	SIR_0113	4	5	4	0
41	Uncharacterized protein	T1ZEG5	SIR_1274	4	6	0	3
42	Signal recognition particle protein	T1ZE30	ffh	5	7	0	0
43	Uncharacterized protein	T1ZGC6	SIR_1156	2	5	3	5
44	Mannosyl-glycoprotein endo-beta-N-acetylglucosaminidase	T1ZEU1	SIR_1072	3	6	2	3
45	DNA-directed RNA polymerase subunit beta	T1ZGS7	rpoB	2	5	4	2
46	Surface antigen	T1ZCQ5	SIR_0054	2	6	5	2
47	Putative adhesion protein	T1ZDS8	fszD	5	5	0	2

48	Hyaluronate lyase	T1ZG27	SIR_1547	3	6	3	2
49	Putative collagen adhesin	T1ZHC4	SIR_1805	3	3	5	2
50	C5a peptidase	T1ZFN2	SIR_1402	3	5	2	2
51	Chaperone protein DnaK	T1ZF47	dnaK	2	5	4	0
52	Putative cell-surface antigen I/II	T1ZHQ3	SIR_1675	0	2	4	0
53	Putative glycosyl transferase	T1ZFQ8	SIR_0933	4	5	0	2
54	Uncharacterized protein	T1ZFV5	SIR_1477	4	3	0	3
55	Formate acetyltransferase	T1ZD63	pfl	2	5	0	3
56	Lysozyme	T1ZF98	SIR_1025	7	2	0	2
57	Translation initiation factor IF-2	T1ZGZ1	infB	0	3	5	2
58	Putative alkaline amylopullulanase	T1ZGL9	pulA2	0	5	4	4
59	Putative stress protein	T1ZDD5	SIR_0040	0	3	3	0
60	3-oxoacyl-[acyl-carrier-protein] synthase 2	T1ZH25	fabF	2	7	0	2
61	DNA polymerase III PolC-type	T1ZGA0	polC	3	5	2	0
62	Uncharacterized protein	T1ZGR7-DECOY		2	0	4	0
63	Glutamine synthetase I alpha	T1ZGF6	glnA	0	4	2	5
64	ATP-dependent zinc metalloprotease FtsH	T1ZDD0	ftsH	3	2	0	4
65	LysM domain-containing protein	T1ZHJ6	SIR_1880	4	4	0	0
66	Uncharacterized protein	T1ZEQ3-DECOY		0	0	3	0
67	Putative phosphoribosylformylglycinamidine synthase	T1ZB52	purL	0	7	2	0
68	Glyceraldehyde-3-phosphate dehydrogenase	T1ZCF7	gap	0	7	0	0
69	Pyruvate formate lyase	T1ZF15	SIR_1079	2	5	0	0
70	Transcription-repair-coupling factor	T1ZC42	trcF	0	0	0	3

71	Chromosome partition protein Smc	T1ZDG1	smc	0	4	2	0
72	Cell division ATP-binding protein FtsE	T1ZF37	ftsE	0	2	2	5
73	50S ribosomal protein L18	T1ZFR9	rplR	2	3	0	0
74	Alkyl hydroperoxide reductase subunit F	T1ZGT3	ahpF	2	4	0	0
75	Uncharacterized protein	T1ZCV9-DECOY		0	0	4	0
76	Valine--tRNA ligase	T1ZFZ4	valS	0	4	2	3
77	Phosphoglycerate kinase	T1ZGG2	pgk	0	2	0	0
78	Beta-N-acetylhexosaminidase	T1ZET7	SIR_1067	2	3	2	0
79	30S ribosomal protein S10	T1ZH81	rpsJ	2	3	0	3
80	GRAM_POS_ANCHORING domain-containing protein	T1ZEJ8	SIR_0758	3	0	0	2
81	Elongation factor G	T1ZDS4	fusA	2	3	0	3
82	Lysine--tRNA ligase	T1ZFC7	lysS	3	2	0	0
83	Putative recombinase	T1ZEH5	SIR_0971	4	2	0	2
84	ABC transporter, substrate-binding protein	T1ZD17	msmE	0	3	0	0
85	Putative conjugal transfer protein	T1ZFW8	SIR_0990	2	6	0	3
86	Pyruvate kinase	T1ZEN4	pyk	0	8	0	0
87	Uncharacterized protein	T1ZCC8	SIR_0176	2	3	0	0
88	DD-transpeptidase	T1ZBB4	SIR_0124	3	3	2	0
89	DUF4366 domain-containing protein	T1ZDN0	SIR_0987	0	2	3	0
90	Type I restriction enzyme R Protein	T1ZDQ2	hsdR	3	4	2	0
91	Putative DNA-entry endonuclease	T1ZDT4	endA	2	3	0	0
92	Putative penicillin binding protein 2B	T1ZDK4	pbp2b	4	0	0	3
93	Threonine--tRNA ligase	T1ZFI3	thrS	3	3	0	0

94	DUF4832 domain-containing protein	T1ZGK3	SIR_1591	0	4	4	0
95	Conjugal transfer protein	T1ZEL6	SIR_1329	4	0	0	0
96	Uncharacterized protein	T1ZDP3	SIR_0613	0	3	6	0
97	Phosphoenolpyruvate-protein phosphotransferase	T1ZG24	ptsI	2	2	0	2
98	Isopentenyl-diphosphate delta-isomerase	T1ZD59	fni	2	5	0	0
99	Peptidyl-prolyl cis-trans isomerase	T1ZE70	ppiA	0	8	0	0
100	Histidine triad protein	T1ZEB0	SIR_0654	0	0	2	2
