# Supplementary figures and images for: Dual Targeting Factors Are Required for LXG Toxin Export by the Bacterial Type VIIb Secretion System
Source: mBio. 2022 Aug 29;13(5):e02137-22. doi: 10.1128/mbio.02137-22 (PMC9600955; doi:10.1128/mbio.02137-22)

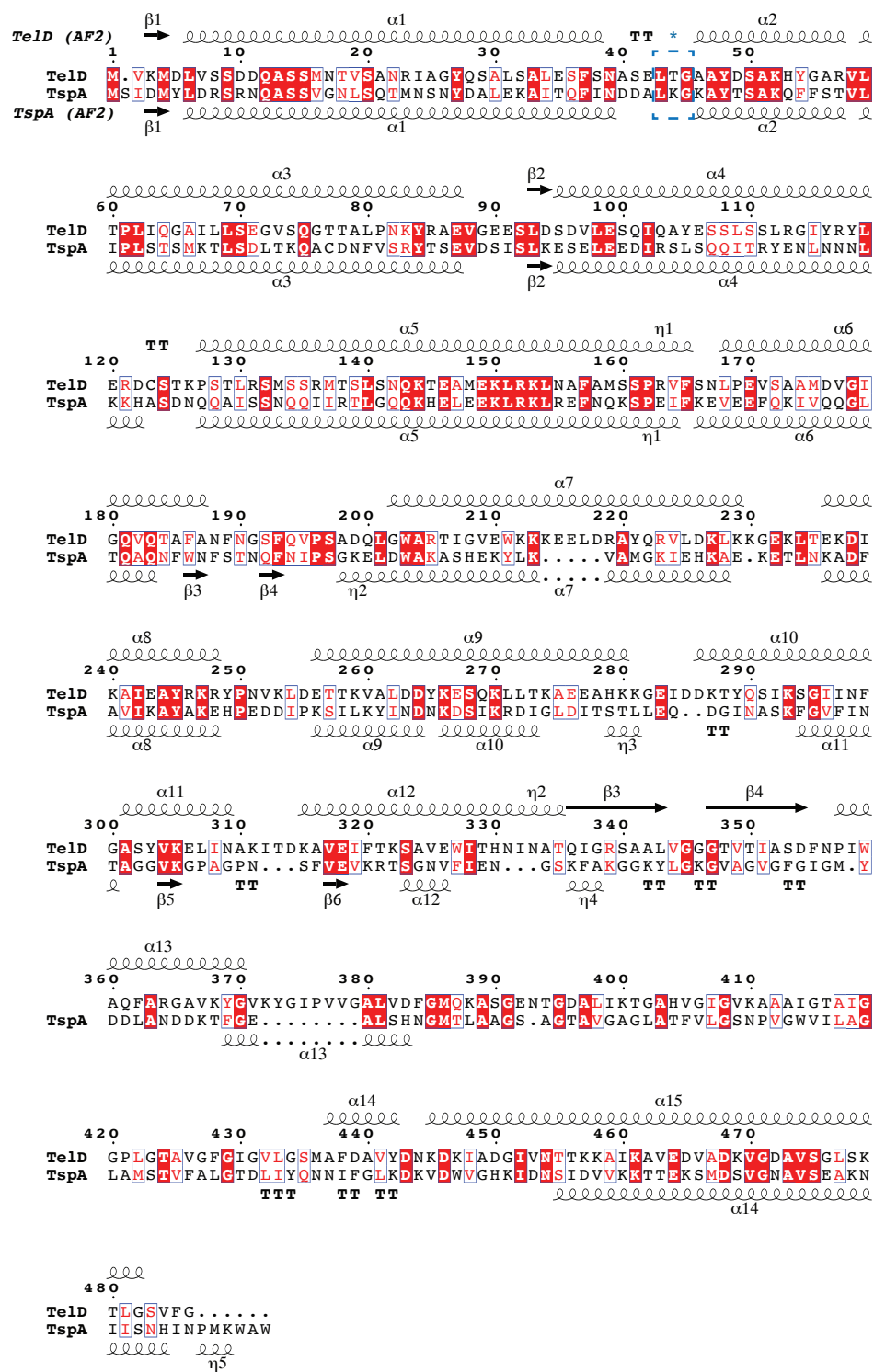

Supplement: FIG S2 [file mbio.02137-22-s0002.pdf]

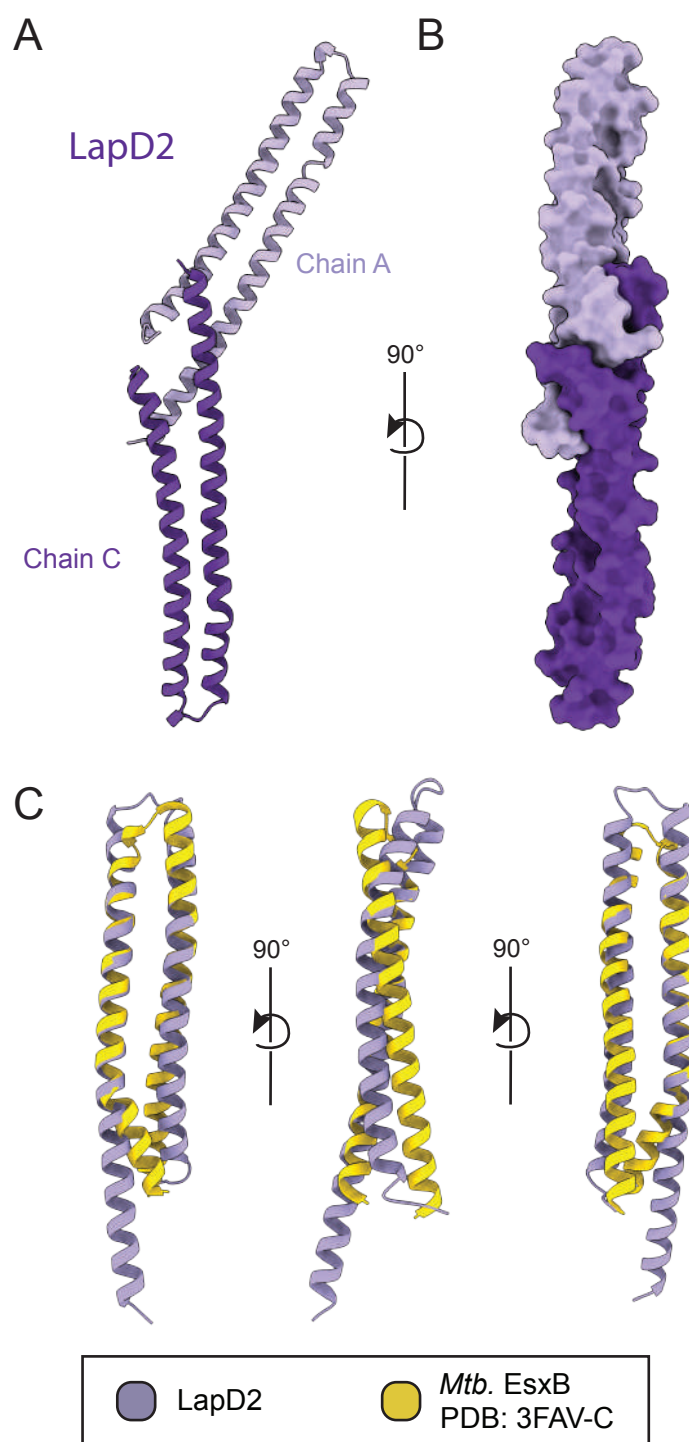

Supplement: FIG S3 [file mbio.02137-22-s0003.pdf]

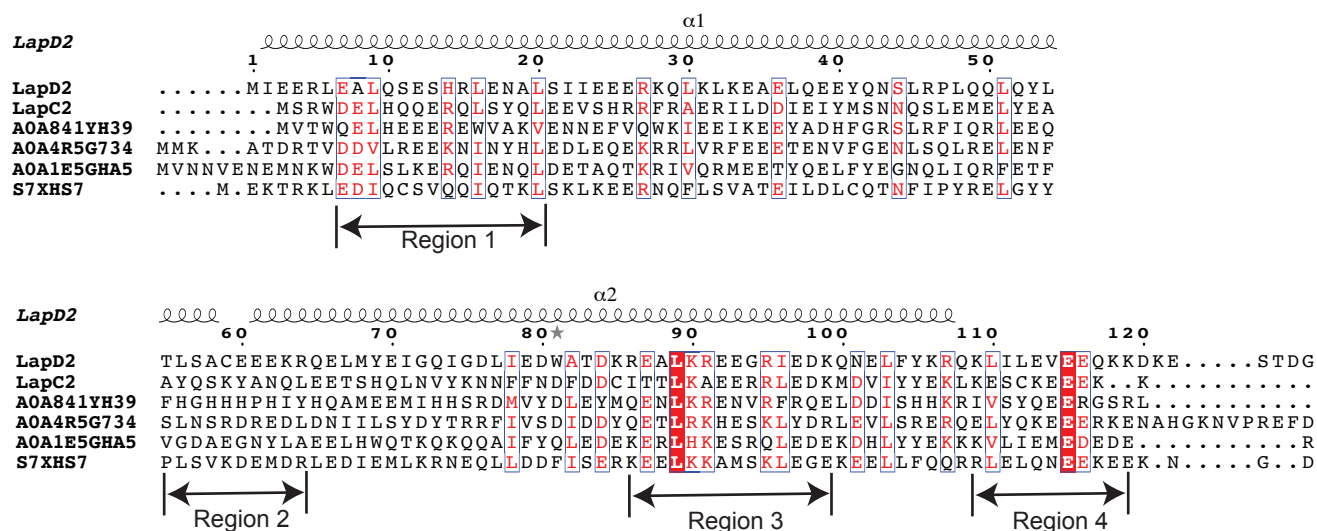

# B

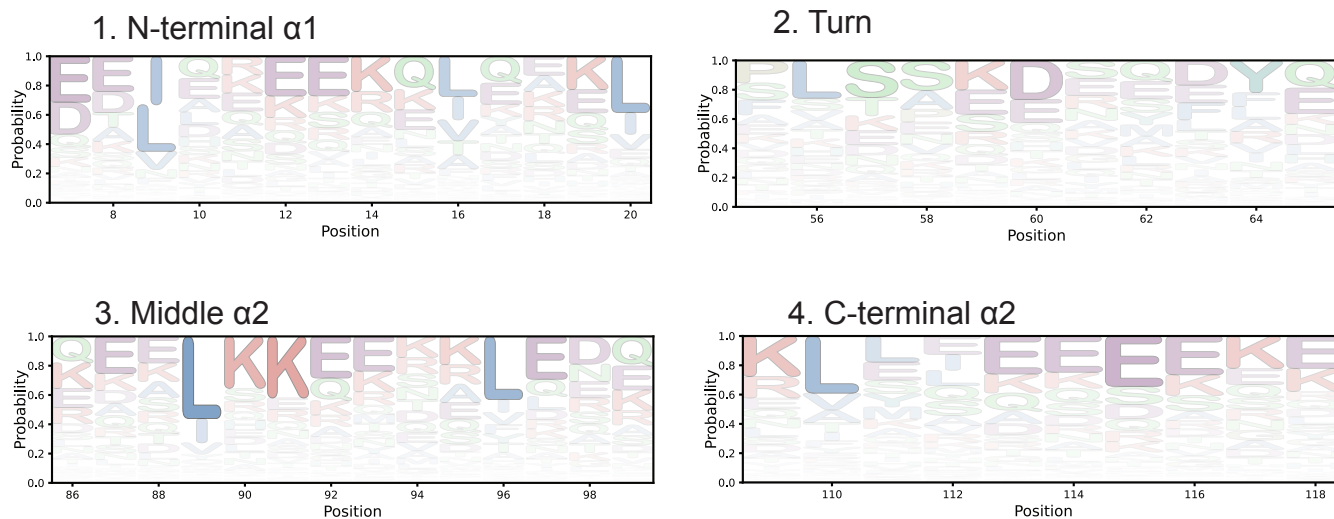

Supplement: FIG S4 [file mbio.02137-22-s0004.pdf]

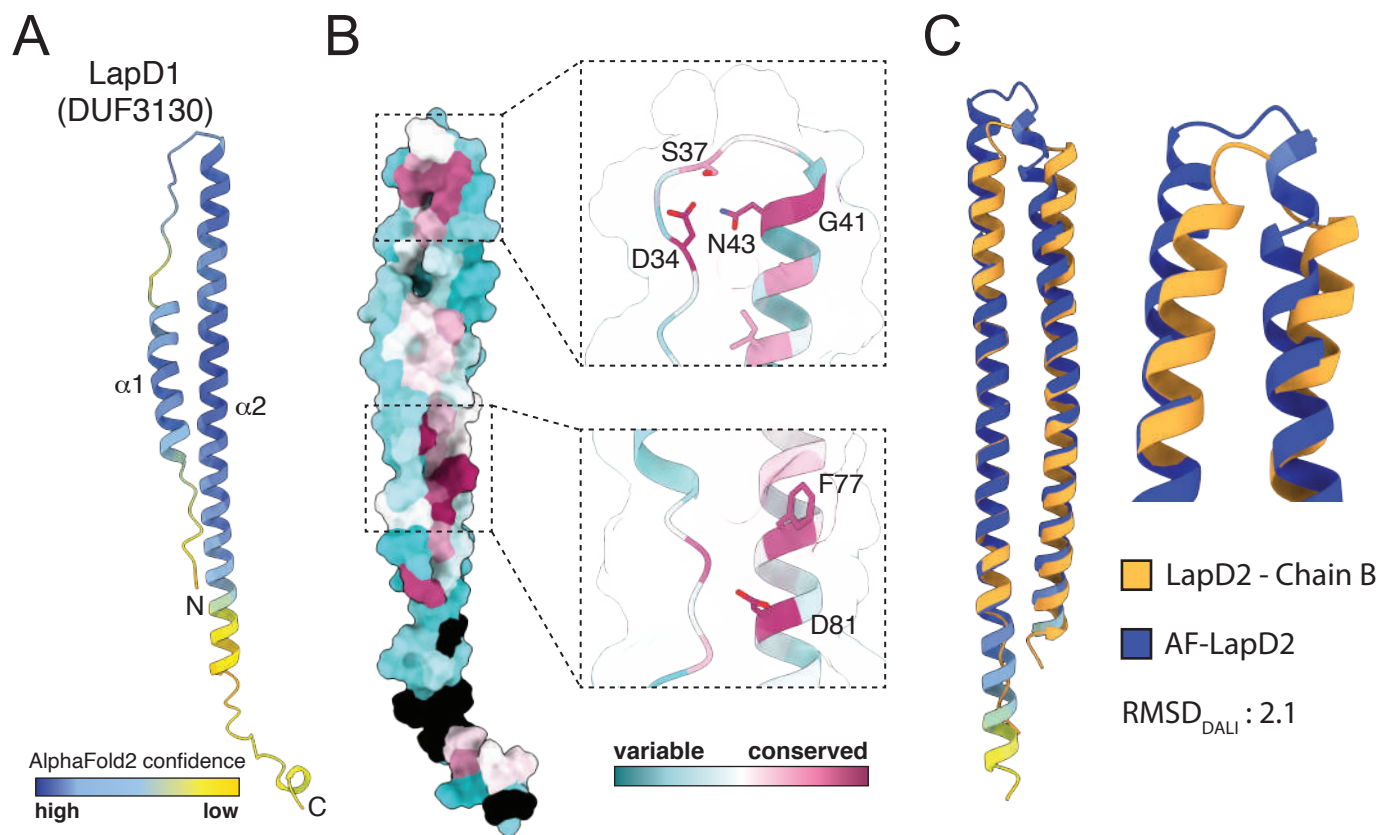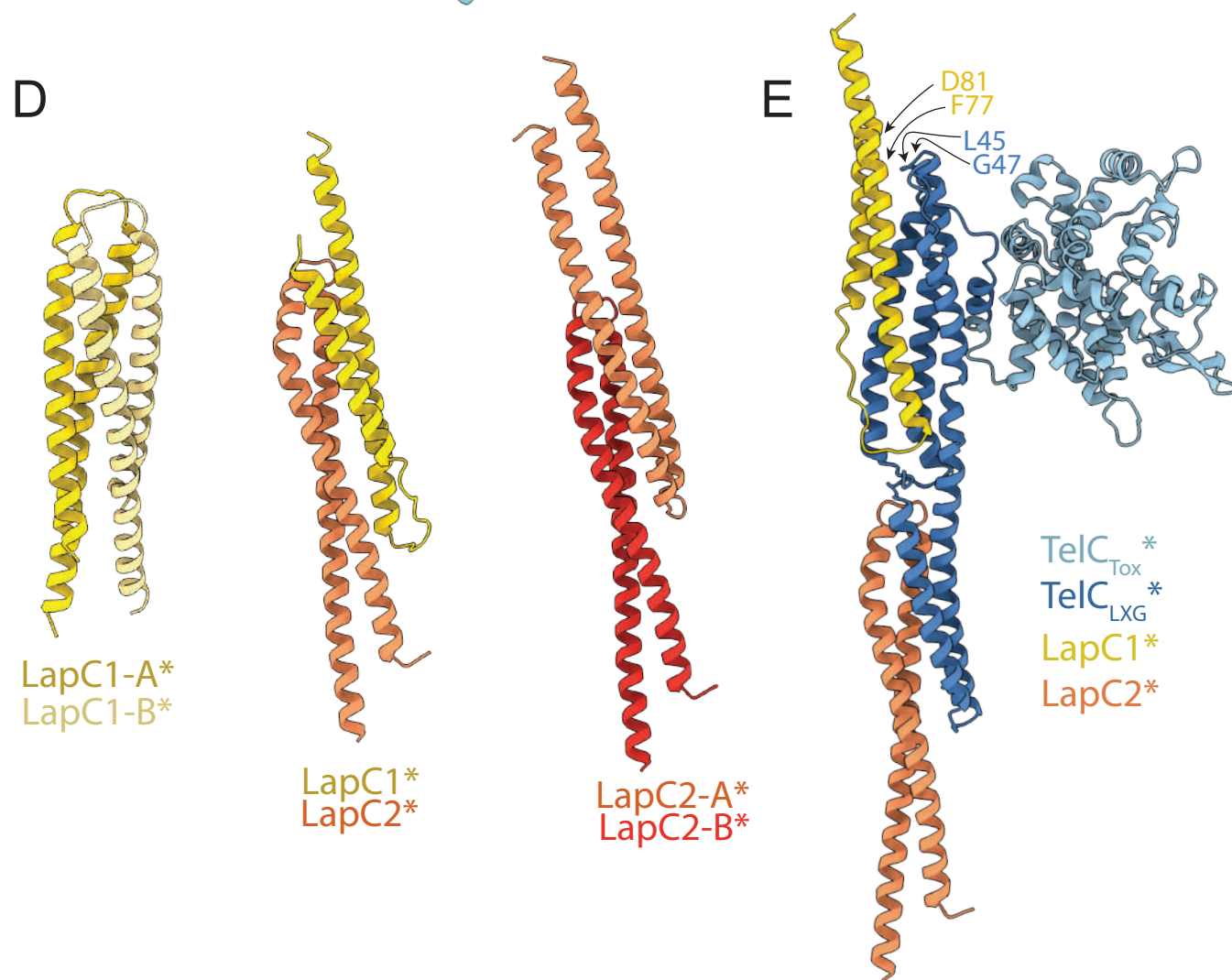

Supplement: FIG S5 [file mbio.02137-22-s0005.pdf]

A

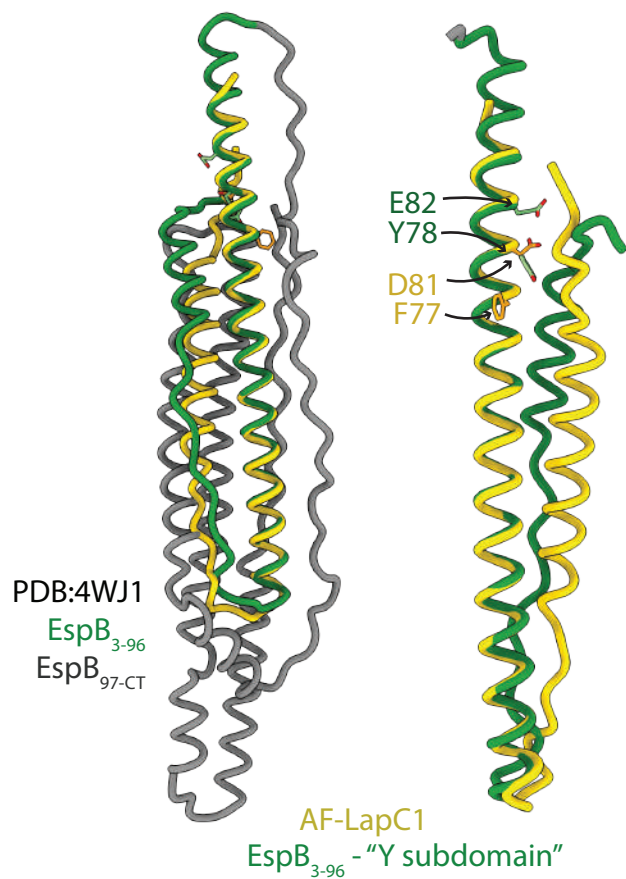

B

PDB:4W4L  
PE25  
PPE41  
EspG5

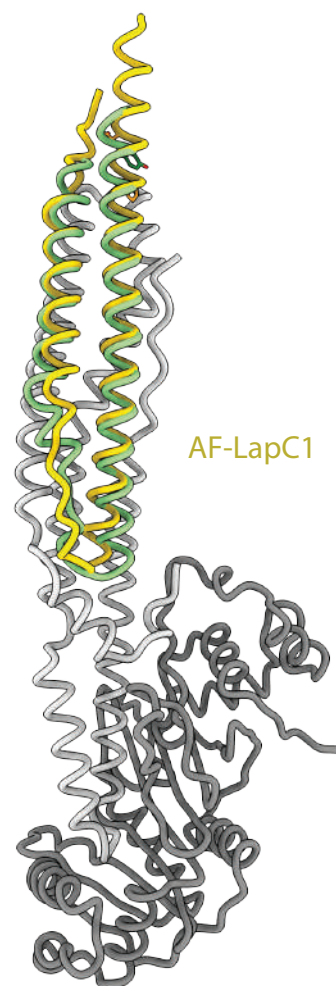

C

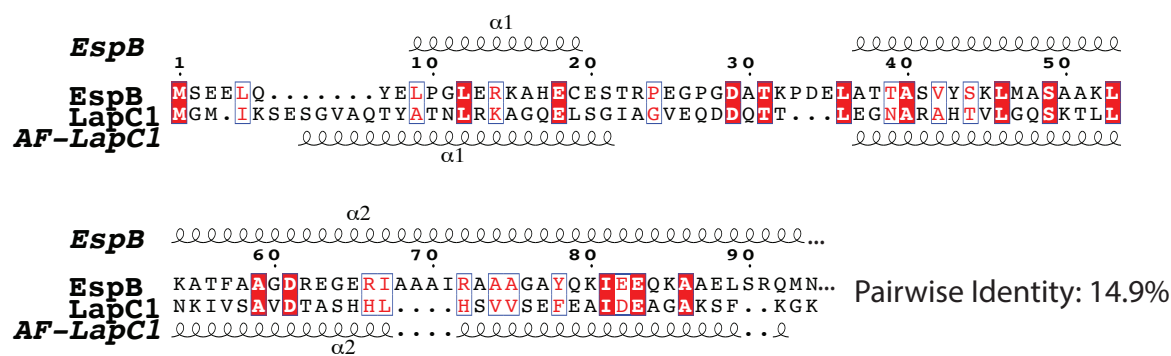

D

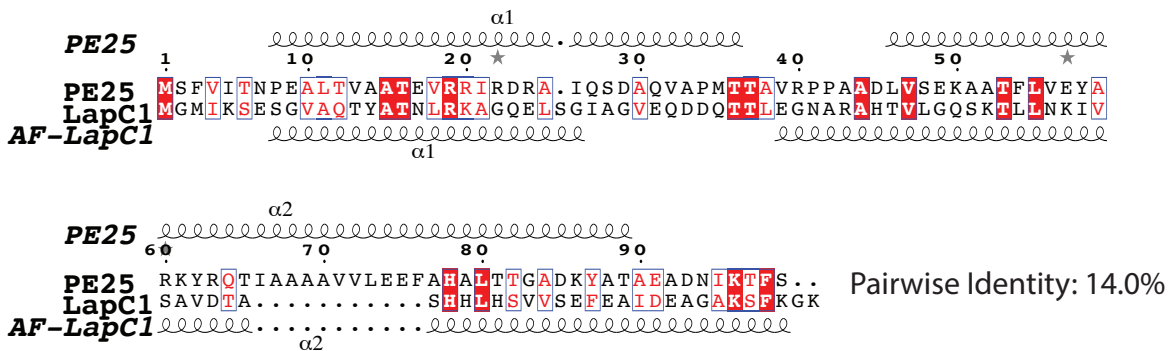

Supplement: FIG S6 [file mbio.02137-22-s0006.pdf]
