# Supplementary material for: Dual Targeting Factors Are Required for LXG Toxin Export by the Bacterial Type VIIb Secretion System
Source: mBio. 2022 Aug 29;13(5):e02137-22. doi: 10.1128/mbio.02137-22 (PMC9600955; doi:10.1128/mbio.02137-22)
Supplement: TABLE S2 [file mbio.02137-22-s0008.pdf]

Table S2.1. Accession codes and sequence information for LapD2 homologs identified with three iterations of JackHMMER.

Entry	Protein names	Gene names	Organism	Length
F0ISI0	Uncharacterized protein	HMPREF9384_0792	Streptococcus sanguinis SK160	120
A0A829IC82	Uncharacterized protein	SAG0014_09635	Streptococcus agalactiae FSL S3-586	120
A0A427Z096	Uncharacterized protein	D8894_04900	Streptococcus oralis	120
F8DHG2	Uncharacterized protein	HMPREF0833_11762	Streptococcus parasanguinis ATCC 15912	118
A0A8B1YUD9	DUF3958 family protein	J4854_01605	Streptococcus lactarius	118
A0A178KGP4	Uncharacterized protein	A3Q39_01935	Streptococcus sp. CCUG 49591	118
A0A1X1IMY3	Uncharacterized protein	B7710_01130	Streptococcus oralis subsp. oralis	120
A0A3R9HBG1	Uncharacterized protein	D8875_04300	Streptococcus sanguinis	120
A3CR32	Uncharacterized protein	SSA_2275	Streptococcus sanguinis (strain SK36)	121
A0A178KI83	Uncharacterized protein	A3Q39_01965	Streptococcus sp. CCUG 49591	124
S7XHS7	Uncharacterized protein	M059_05495	Streptococcus mitis 18/56	124
A0A139P9I6	Uncharacterized protein	SORDD16_01672	Streptococcus oralis	121
A0A3R9JF83	Uncharacterized protein	D8839_01325	Streptococcus mitis	118
A0A428A3Y3	Uncharacterized protein	D8883_04735	Streptococcus sanguinis	120
A0A427ZT62	Uncharacterized protein	D8886_05325	Streptococcus sanguinis	120
A0A5A7ZT25	Uncharacterized protein	FKX92_00600	Streptococcus sanguinis	129
A0A7H8V963	Uncharacterized protein	FFV08_11455	Streptococcus sanguinis	120
A0A123VUG4	FKBP_N domain-containing protein	ERS132372_01528 ERS132399_02391	Streptococcus suis	128

A0A428A688	Uncharacterized protein	D8879_11740	Streptococcus sanguinis	120
A0A1F0ZSH8	Uncharacterized protein	HMPREF2917_09360	Streptococcus sp. HMSC061E03	118
F3UNP6	Uncharacterized protein	HMPREF9389_0454	Streptococcus sanguinis SK355	121
A0A1X1JWY6	Uncharacterized protein	B7700_09660	Streptococcus mitis	118
A0A345VJJ3	Uncharacterized protein	Sp14A_09740	Streptococcus pluranimalium	129
A0A8B4IQ53	Uncharacterized protein	NCTC3858_00393	Streptococcus uberis	122
A0A0F5MM48	Uncharacterized protein	RN86_02675	Streptococcus gordonii	132
A0A0F2CF76	Uncharacterized protein	TZ86_01640 UA00_00089	Streptococcus gordonii	119
A0A2X3XZG6	Uncharacterized protein	NCTC12278_01112	Streptococcus ferus	131
A0A0E1EH98	Uncharacterized protein	AX245_04160 C4618_11680 C6N07_05900 RDF_1029	Streptococcus agalactiae	118
A0A4T2H8W2	Uncharacterized protein	FAJ36_02910	Streptococcus suis	123
A0A1V0H1D1	Uncharacterized protein	A6J85_03500	Streptococcus gordonii	118
A0A7H8UYG8	Energy transducer TonB	FDP16_01525	Streptococcus sanguinis	120
A0A1E5GHA5	Uncharacterized protein	BCR21_07310	Enterococcus ureasiticus	126
A0A1X1J4E5	Uncharacterized protein	B7708_00960	Streptococcus oralis subsp. dentisani	124
A0A7Z0VFP3	Uncharacterized protein	TH70_0121	Streptococcus agalactiae	123
A0A1E5HGJ1	Uncharacterized protein	BCR24_01620	Enterococcus ureilyticus	118
A0A4P7WQS8	Uncharacterized protein	E8M06_09955 E8M06_09985	Streptococcus suis	123
A0A0U2NRK3	Uncharacterized protein	ATZ35_10685	Enterococcus rotai	118

E6KIR2	Uncharacterized protein	HMPREF8578_0127	Streptococcus oralis ATCC 49296	120
A0A4R5G734	Uncharacterized protein	E0E04_02155	Streptococcus vicugnae	134
A0A6I3PB65	Uncharacterized protein	GMC80_04755 GMC84_06710	Streptococcus parasanguinis	118
A0A7X2UEL6	Uncharacterized protein	NCTC3858_01463	Streptococcus uberis	126
E6KIP9	Uncharacterized protein	HMPREF8578_0114	Streptococcus oralis ATCC 49296	118
A0A540UNN3	FKBP_N domain-containing protein	FH692_10965	Streptococcus suis	128
A0A3L8GE13	Uncharacterized protein	DIY07_08810	Streptococcus iniae (Streptococcus shiloi)	125
A0A7H9FG12	Uncharacterized protein	HRE59_00315	Streptococcus oralis subsp. oralis	118
A0A372KJ05	Uncharacterized protein	DDV21_010945 DDV23_10765	Streptococcus chenjunshii	131
A0A0J6KU02	Uncharacterized protein	VK90_24155	Bacillus sp. LK2	116
A0A427Z4E3	Energy transducer TonB	D8889_08515 FKX92_06260	Streptococcus sanguinis	120
A0A0F5MJX1	Uncharacterized protein	RN86_02700	Streptococcus gordonii	118
A0A7X2UQ75	Uncharacterized protein	NCTC3858_01475	Streptococcus uberis	126
A0A0S3K6Z3	Uncharacterized protein	ATZ33_01285	Enterococcus silesiacus	118
A0A242AUF8	Uncharacterized protein	A5821_000622	Enterococcus sp. 7F3_DIV0205	120
A0A242H4J5	Uncharacterized protein	A5866_002132	Enterococcus sp. 12C11_DIV0727	118
A0A242CWU2	Uncharacterized protein	A5875_003888	Enterococcus sp. 3H8_DIV0648	119
F0IN33	HD domain protein	HMPREF9383_1536	Streptococcus sanguinis SK150	119
A0A427ZN60	Uncharacterized protein	D8886_09175	Streptococcus sanguinis	120
A0A081QRU4	Cell-cycle control medial ring	SK578_0511	Streptococcus mitis	124

	component family protein			
A0A242ATT0	Uncharacterized protein	A5821_000410	Enterococcus sp. 7F3_DIV0205	119
A0A3R9J4D5	Uncharacterized protein	D8860_09785	Streptococcus oralis	118
A0A2X3VDB5	Uncharacterized protein	NCTC11085_00303	Streptococcus sanguinis	120
A0A1X1HW15	Uncharacterized protein	B7714_09145	Streptococcus oralis subsp. oralis	120
A0A0Z8JBB1	Uncharacterized protein	ERS132440_00897	Streptococcus suis	123
A0A242GZP3	Uncharacterized protein	A5866_000650	Enterococcus sp. 12C11_DIV0727	114
A0A841YH39	DUF3958 family protein	HB844_13135	Listeria fleischmannii	118
A0A1E5GX96	Uncharacterized protein	BCR23_04630	Enterococcus quebecensis	115
R2T5H1	Uncharacterized protein	UAY_00975	Enterococcus moraviensis ATCC BAA-383	118
A0A7H8V9W6	Uncharacterized protein	FFV08_11490	Streptococcus sanguinis	120
F0FHF6	Uncharacterized protein	HMPREF9388_2139	Streptococcus sanguinis SK353	121
A0A1X1IPR0	Uncharacterized protein	B7710_00060	Streptococcus oralis subsp. oralis	118
A0A428G5R6	Uncharacterized protein	D8801_04900	Streptococcus oralis	124
A0A0N0KTL2	Uncharacterized protein	AEQ18_02380	Enterococcus sp. RIT-PI-f	116
A0A200JBQ9	Uncharacterized protein	A5889_000138	Enterococcus sp. 9D6_DIV0238	117
A0A7D4GRI0	Uncharacterized protein	FOC63_06870	Streptococcus gallolyticus	134
A0A4T2GM54	Uncharacterized protein	FAJ39_07710	Streptococcus suis	128
A0A242LA88	Uncharacterized protein	A5881_003618	Enterococcus termitis	118
A0A380IM03	Uncharacterized protein	NCTC6175_01411	Streptococcus agalactiae	120

A0A4V6U7E4	Uncharacterized protein	FAJ36_02880	Streptococcus suis	128
A0A3R9HGP9	Uncharacterized protein	D8887_07705	Streptococcus sanguinis	113
A0A7Z7QUJ7	Uncharacterized protein	NCTC8183_01312	Streptococcus agalactiae	133
A0A139NND7	Uncharacterized protein	STRDD11_02626	Streptococcus sp. DD11	120
A0A2L0D3F4	Uncharacterized protein	C0J00_04050	Streptococcus pluranimalium	131
R2T9D5	Uncharacterized protein	UAY_02590	Enterococcus moraviensis ATCC BAA-383	117
A0A0Z8HRE2	Uncharacterized protein	ERS132406_02094	Streptococcus suis	123
A0A0B7GNC7	Uncharacterized protein	SSV_1920	Streptococcus sanguinis	120
F9LWN3	Uncharacterized protein	HMPREF9965_0736	Streptococcus mitis bv. 2 str. SK95	118
A0A1X1JX79	Uncharacterized protein	B7700_09690	Streptococcus mitis	124
A0A1E5H5L3	Uncharacterized protein	BCR24_09885	Enterococcus ureilyticus	122
F0IBB8	Uncharacterized protein	HMPREF9382_2056	Streptococcus sanguinis SK115	120
R3W643	Uncharacterized protein	UC3_02024	Enterococcus phoeniculicola ATCC BAA-412	116
A0A428IHC8	Uncharacterized protein	D8844_06490	Streptococcus oralis	120
A0A2W4BKR6	Uncharacterized protein	CI088_09485	Enterococcus plantarum	115
A0A3R9H620	Uncharacterized protein	D8879_10595	Streptococcus sanguinis	120
A0A1E5GJU3	Uncharacterized protein	BCR25_08215	Enterococcus termitis	115
A0A0Z8I4W5	Uncharacterized protein	ERS132410_02192	Streptococcus suis	123

Table S2.2. Accession codes and sequence information for LapC1 homologs identified with one iteration of JackHMMER.

Entry	Protein names	Gene names	Organism	Length
T1ZH75	Uncharacterized protein	SIR_1491	Streptococcus intermedius B196	91
A0A0E2IQB7	Uncharacterized protein	HMPREF1654_01870	Streptococcus intermedius ATCC 27335	91
A0A139R5L5	TIGR04197 family type VII secretion effector	FOC63_00900 SGADD02_00470 SGADD03_00389	Streptococcus gallolyticus	93
A0A1S5WDW5	Uncharacterized protein	BTR42_08900	Streptococcus gallolyticus subsp. gallolyticus DSM 16831	93
A0A1I7GQI7	Type VII secretion effector, SACOL2603 family	SAMN05660328_102271	Streptococcus gallolyticus	93
F5WVX6	Uncharacterized protein	SGGB_1575	Streptococcus gallolyticus ATCC 43143	93
E8K2Z3	Uncharacterized protein	HMPREF9423_1856	Streptococcus infantis ATCC 700779	92
A0A1H8Z4E7	Type VII secretion effector, SACOL2603 family	SAMN05216346_101162	Streptococcus equinus (Streptococcus bovis)	90
A0A139QYV5	Uncharacterized protein	SGADD02_00817 SGADD03_01202	Streptococcus gallolyticus	90
F9LY30	Uncharacterized protein	HMPREF9965_1762	Streptococcus mitis bv. 2 str. SK95	92
A0A1C3SMV2	Uncharacterized protein	SMA679_0761	Streptococcus macedonicus	90
A0A3R9HJH6	Uncharacterized protein	D8863_08620	Streptococcus oralis	92
A0A1H0MTA7	Type VII secretion effector, SACOL2603 family	SAMN05216347_102469	Streptococcus equinus (Streptococcus bovis)	90

A0A371QFB0	TIGR04197 family type VII secretion effector	DXN33_01140	Streptococcus sp. NM	92
A0A3R9QBN4	Uncharacterized protein	D8786_05750 D8855_04310	Streptococcus mitis	92
A0A1F0BUA5	Type VII secretion protein	HMPREF2613_07245	Streptococcus sp. HMSC070B10	92
A0A501PB50	TIGR04197 family type VII secretion effector	FJN11_06485	Streptococcus symci	92
A0A3R9HQE0	TIGR04197 family type VII secretion effector	D8789_07065 D8849_09150 D8865_10365 JJN14_03035	Streptococcus mitis	92
A0A1E9GAV6	Type VII secretion protein	HMPREF2766_03755	Streptococcus sp. HMSC076C08	92
A0A2G3NUY4	TIGR04197 family type VII secretion effector	CS009_05415 CS010_03220	Streptococcus macedonicus	90
A0A7D4GS34	TIGR04197 family type VII secretion effector	FOC63_08560	Streptococcus gallolyticus	90
A0A1S5WBI2	Uncharacterized protein	BTR42_04595	Streptococcus gallolyticus subsp. gallolyticus DSM 16831	90
A0A1B1ID96	Type VII secretion protein	AXF18_01730	Streptococcus sp. oral taxon 064	92
A0A2I1UMC7	TIGR04197 family type VII secretion effector	CYK17_09995	Streptococcus oralis subsp. dentisani	92
A0A1S0ZA19	Type VII secretion protein	A7T00_33115	Salmonella enterica subsp. enterica serovar Saintpaul	92
A0A380K862	Type VII secretion effector	NCTC13767_01892	Streptococcus gallolyticus	90
A0A1H6SD36	Type VII secretion	SAMN05216460_1192	Streptococcus sp. 45	90

	effector, SACOL2603 family			
A0A3R9FX19	Uncharacterized protein	D8894_04980	Streptococcus oralis	92
A0A1X1J9V7	Type VII secretion effector	B7705_06215	Streptococcus oralis subsp. dentisani	92
A0A428DJZ0	Uncharacterized protein	D8847_09950	Streptococcus mitis	92
A0A3R9J234	Uncharacterized protein	D8847_09775	Streptococcus mitis	92
F5X0A7	Uncharacterized protein	SGGB_0839	Streptococcus gallolyticus ATCC 43143	90
A0A139PV09	Uncharacterized protein	SORDD27_01490	Streptococcus oralis	92
I0Q5G0	Type VII secretion effector, TIGR04197 family	HMPREF1115_1692	Streptococcus oralis SK610	92
A0A1I7FJ84	Type VII secretion effector, SACOL2603 family	SAMN05660328_101420	Streptococcus gallolyticus	90
A0A239RBG6	Type VII secretion effector, SACOL2603 family	SAMN05216470_0920	Streptococcus equinus (Streptococcus bovis)	90
A0A081QNZ9	Uncharacterized protein	SK578_0768 SMIM3I_00648 SMIM3IV_00595	Streptococcus mitis	92
A0A231VWK6	TIGR04197 family type VII secretion effector	CBI42_08510	Streptococcus sp. KR	92
A0A1F0B683	Type VII secretion protein	HMPREF2701_04775	Streptococcus sp. HMSC077D04	92
A0A4V0BUI7	Type VII secretion effector	NCTC5338_01391	Streptococcus australis	92

A0A4V6LQ02	Type VII secretion effector	NCTC10232_01364	Streptococcus oralis	92
A0A2X3W4X4	Type VII secretion effector	NCTC12278_01169	Streptococcus ferus	91
A0A1X1INN9	Type VII secretion effector	B7710_01210	Streptococcus oralis subsp. oralis	92
A0A3R9KT57	Uncharacterized protein	D8788_09675	Streptococcus mitis	92
J5H474	Type VII secretion effector, TIGR04197 family	HMPREF1125_0309	Streptococcus oralis SK304	92
A0A1S1CRP1	Type VII secretion protein	HMPREF2628_07975	Streptococcus sp. HMSC063B03	92
A0A139QMH4	Uncharacterized protein	SORDD24_01549	Streptococcus oralis	92
A0A1X1H983	Type VII secretion effector	B7721_02930	Streptococcus oralis subsp. oralis	92
A0A428IP91	Uncharacterized protein	D8846_06225	Streptococcus oralis	92
A0A1X1HPW8	Type VII secretion effector	B7716_01660	Streptococcus oralis subsp. oralis	92
E9FIV1	Uncharacterized protein	HMPREF0849_01627	Streptococcus sp. C300	92
A0A139Q4A6	Type VII secretion protein	BBP19_06505 SORDD30_01629	Streptococcus oralis	92
A0A1X1GSZ3	Type VII secretion effector	B7712_00855	Streptococcus oralis subsp. oralis	92
A0A139M8G1	Uncharacterized protein	SORDD05_01233	Streptococcus oralis	92
A0A139QLX9	Uncharacterized protein	SORDD24_01677	Streptococcus oralis	92
A0A139PVN7	Uncharacterized protein	D8844_06410 SORDD20_00506	Streptococcus oralis	92

A0A1X1HNL4	Type VII secretion effector	B7718_02130	Streptococcus oralis subsp. oralis	92
A0A428HB07	Uncharacterized protein	D8788_03670	Streptococcus mitis	92
G6C8X2	Uncharacterized protein	HMPREF9184_00751	Streptococcus sp. oral taxon 058 str. F0407	92
A0A4Q2FKS1	TIGR04197 family type VII secretion effector	DF216_07805	Streptococcus oralis	92
J5GN34	Type VII secretion effector, TIGR04197 family	HMPREF1125_2061	Streptococcus oralis SK304	92
A0A1X0X0B5	Type VII secretion protein	ATE37_07430	Streptococcus oralis subsp. tigurinus	92
A0A428CAR2	Uncharacterized protein	D8856_09625	Streptococcus mitis	92
A0A3R9KGB9	Uncharacterized protein	D8854_03060	Streptococcus mitis	92
A0A1X1I2K2	Type VII secretion effector	B7714_02825	Streptococcus oralis subsp. oralis	92
A0A139NUT4	Uncharacterized protein	SORDD14_01568	Streptococcus oralis	92
A0A4Q2FL97	TIGR04197 family type VII secretion effector	DF216_07290	Streptococcus oralis	92
A0A1X1H062	Type VII secretion effector	B7722_01935	Streptococcus oralis subsp. oralis	92
A0A4R5G4Y4	TIGR04197 family type VII secretion effector	E0E04_04080	Streptococcus vicugnae	91
A0A1X1GBF8	Type VII secretion effector	B7727_03280	Streptococcus oralis subsp. tigurinus	93
A0A139NWT6	Uncharacterized protein	SORDD15_01377	Streptococcus oralis	92

A0A135YLC9	Uncharacterized protein	HMPREF3205_02308	Streptococcus pasteurianus	96
A0A1S5WCM9	Uncharacterized protein	BTR42_05645	Streptococcus gallolyticus subsp. gallolyticus DSM 16831	91
A0A7D4GHM0	TIGR04197 family type VII secretion effector	FOC63_09620	Streptococcus gallolyticus	91
A0A7D4K0Q8	TIGR04197 family type VII secretion effector	FOC63_07845	Streptococcus gallolyticus	91
A0A1I7F6C6	Type VII secretion effector, SACOL2603 family	SAMN05660328_101220	Streptococcus gallolyticus	91
A0A1I7FC93	Type VII secretion effector, SACOL2603 family	SAMN05660328_101314	Streptococcus gallolyticus	91
A0A139NQ79	Uncharacterized protein	STRDD11_02464	Streptococcus sp. DD11	89
F3USM5	Uncharacterized protein	HMPREF9389_1833	Streptococcus sanguinis SK355	90
A0A3R9IAM7	TIGR04197 family type VII secretion effector	D8887_08455 FFV08_05580	Streptococcus sanguinis	90
A0A427ZP46	Uncharacterized protein	D8886_07895	Streptococcus sanguinis	90
A0A3R9NTY4	Uncharacterized protein	D8879_08845	Streptococcus sanguinis	90
G5JR52	Uncharacterized protein	STRCR_1677 STRCR_1937	Streptococcus criceti HS-6	92
A0A2A5SDM4	TIGR04197 family type VII secretion effector	FEZ46_05180 RU88_GL002128	Lactococcus raffinolactis	102
A0A0F3H405	Uncharacterized protein	TZ97_00642	Streptococcus parasanguinis	89

A0A6N3CT23	Uncharacterized protein	SPLFYP13_01158	Streptococcus parasanguinis	89
F8DGG8	Uncharacterized protein	HMPREF0833_10386	Streptococcus parasanguinis ATCC 15912	94
A0A359YGK2	Uncharacterized protein	SPADD19_01110	Streptococcus parasanguinis	89
A0A1F1A3X5	Uncharacterized protein	HMPREF2917_04405	Streptococcus sp. HMSC061E03	89
I1ZLJ5	Uncharacterized protein	Spaf_0919	Streptococcus parasanguinis FW213	103
A0A4Q5BT34	TIGR04197 family type VII secretion effector	GMC84_09185 GMC94_02205	Streptococcus parasanguinis	89
I2NMG3	Uncharacterized protein	HMPREF9971_1232	Streptococcus parasanguinis F0449	113
G5JRP1	Uncharacterized protein	STRCR_2050	Streptococcus criceti HS-6	91
A0A1F0AWW4	Uncharacterized protein	HMPREF2686_08175	Streptococcus sp. HMSC057G03	89
V8BGZ4	Uncharacterized protein	HMPREF1195_00404	Streptococcus parasanguinis CC87K	89
A0A428B5A9	Uncharacterized protein	D8866_01720	Streptococcus parasanguinis	89
A0A4Q2FH31	TIGR04197 family type VII secretion effector	DF218_03565	Streptococcus parasanguinis	89
A0A6I3PR01	TIGR04197 family type VII secretion effector	GMC95_02245	Streptococcus parasanguinis	94
E8K4F1	Uncharacterized protein	HMPREF8577_0436	Streptococcus parasanguinis ATCC 903	99
A0A6A0B9J2	Type VII secretion protein	Hs30E_00170	Lactococcus hodotermopsidis	101
E3CE17	Uncharacterized protein	HMPREF9626_1164	Streptococcus parasanguinis F0405	89
A0A4R5G605	TIGR04197 family type VII	E0E04_02150	Streptococcus vicugnae	118

	secretion effector			
A0A7D4GGP0	TIGR04197 family type VII secretion effector	FOC63_06865	Streptococcus gallolyticus	118
A0A139MU55	Uncharacterized protein	STRDD04_00268	Streptococcus sp. DD04	97
R2R504	Type VII secretion effector	UAI_02685	Enterococcus malodoratus ATCC 43197	92
A0A8B1YTF9	TIGR04197 family type VII secretion effector	J4854_05255	Streptococcus lactarius	89
A0A224XFQ7	Uncharacterized protein	RsY01_1995	Lactococcus reticulitermitis	102
A0A0A0DFU7	Uncharacterized protein	SSIN_0557	Streptococcus sinensis	104
A0A242DHL2	Uncharacterized protein	A5875_002996	Enterococcus sp. 3H8_DIV0648	92
A0A7W1YHC9	TIGR04197 family type VII secretion effector	HPK16_15390	Listeria rustica	91
A0A378MC82	Type VII secretion effector	NCTC10815_01240	Listeria grayi (Listeria murrayi)	92
A0A0S3K6Z8	Uncharacterized protein	ATZ33_01280	Enterococcus silesiacus	95
A0A0U2XFK1	Uncharacterized protein	ATZ35_10680	Enterococcus rotai	95
A0A242H2N4	Uncharacterized protein	A5866_002133	Enterococcus sp. 12C11_DIV0727	95
A0A1E5KVA8	Uncharacterized protein	BCR26_15430	Enterococcus rivorum	93
R2TRA5	Type VII secretion effector	UAY_00974	Enterococcus moraviensis ATCC BAA-383	95
D7V0H1	Uncharacterized protein	HMPREF0556_11749	Listeria grayi DSM 20601	95
K8N1C5	Uncharacterized protein	HMPREF9186_00129	Streptococcus sp. F0442	89

A0A242L9F8	Uncharacterized protein	A5881_003619	Enterococcus termitis	95
A0A1E5HGJ2	Uncharacterized protein	BCR24_01625	Enterococcus ureilyticus	95
A0A2R7ZZP2	Uncharacterized protein	CDIMF43_180250 CKN86_07930	Carnobacterium divergens (Lactobacillus divergens)	92
A0A830LAN8	TIGR04197 family type VII secretion effector	CW834_00955	Listeria monocytogenes	97
A0A242ATT5	Uncharacterized protein	A5821_000409	Enterococcus sp. 7F3_DIV0205	95
A0A242CX89	Uncharacterized protein	A5875_003889	Enterococcus sp. 3H8_DIV0648	95
A0A842EF80	TIGR04197 family type VII secretion effector	HB895_12440 HCB08_04225 HCB25_04225 HCB35_09535	Listeria booriae	96
A0A7X0XEW8	TIGR04197 family type VII secretion effector	HCI99_14105 HCJ13_00955	Listeria booriae	96
A0A5E9H6J9	Type VII secretion effector	NCTC13772_01143 NCTC13772_02346	Carnobacterium divergens (Lactobacillus divergens)	92
A0A0J6L2B8	Type VII secretion effector	VK90_21625	Bacillus sp. LK2	99
A0A3R9G5C1	Uncharacterized protein	D8887_07710	Streptococcus sanguinis	92
A0A5E9H653	Type VII secretion effector	NCTC13772_02372	Carnobacterium divergens (Lactobacillus divergens)	92
A0A7X0WR26	TIGR04197 family type VII secretion effector	HB856_08660 HCB51_16600	Listeria booriae	96
A0A081QQI2	Uncharacterized protein	D8845_00760 D8855_02220	Streptococcus mitis	102

		D8865_04910 SK578_1302		
A0A428IXG9	Uncharacterized protein	D8800_00795	Streptococcus oralis	102
R0P931	Uncharacterized protein	D065_00650	Streptococcus mitis 13/39	102
A0A0B7GL02	Putative type VII secretion effector	SSV_1220	Streptococcus sanguinis	97
A0A1E5KUF8	Uncharacterized protein	BCR26_04480	Enterococcus rivorum	93
A0A7Z8G2U5	Uncharacterized protein	CKN67_07395	Carnobacterium divergens (Lactobacillus divergens)	92
A0A8B5GW87	Uncharacterized protein	CKN75_08770	Carnobacterium divergens (Lactobacillus divergens)	92
A0A0S3KD07	Uncharacterized protein	ATZ33_12420	Enterococcus silesiacus	117
R2QLU5	Type VII secretion effector	UAY_03088	Enterococcus moraviensis ATCC BAA-383	120
A0A7I0FCU7	Uncharacterized protein	CKN77_09500	Carnobacterium divergens (Lactobacillus divergens)	92
A0A242AQ39	Uncharacterized protein	A5821_003000	Enterococcus sp. 7F3_DIV0205	120
A0A7X9QZ50	TIGR04197 family type VII secretion effector	HF881_01535	Streptococcus sp. WB01_FAA12	102
W7C7M5	Uncharacterized protein	MFLO_05320	Listeria floridensis FSL S10-1187	96
A0A2R8A462	Uncharacterized protein	CDIMF43_50002 CKN69_02300 CKN86_04715	Carnobacterium divergens (Lactobacillus divergens)	92
A0A200JBQ5	Uncharacterized protein	A5889_000137	Enterococcus sp. 9D6_DIV0238	93
A0A6L6HCB9	TIGR04197 family type VII	GIX45_16890	Erwinia sp. CPCC 100877	93

	secretion effector			
A0A0J6L0K0	Type VII secretion effector	VK90_24150	Bacillus sp. LK2	99
F0IBB7	Uncharacterized protein	HMPREF9382_2055	Streptococcus sanguinis SK115	90
A0A346NBA4	TIGR04197 family type VII secretion effector	DDV21_004010 DDV21_004700 DDV23_11140	Streptococcus chenjunshii	93
A0A4R6ZPZ6	Type VII secretion effector (TIGR04197 family)	DFP96_102255	Listeria rocourtiae	96
A0A842AZ36	TIGR04197 family type VII secretion effector	HCJ13_15535	Listeria booriae	102
A0A2C1R825	TIGR04197 family type VII secretion effector	CON44_02325	Bacillus cereus	99
A0A5F0MN81	Uncharacterized protein	CKN67_04115 CKN75_04550	Carnobacterium divergens (Lactobacillus divergens)	92
A0A2W3Z748	TIGR04197 family type VII secretion effector	CI088_09490	Enterococcus plantarum	96
A0A0N0KSY6	Type VII secretion effector	AEQ18_02375	Enterococcus sp. RIT-PI-f	88
A0A7X0T610	TIGR04197 family type VII secretion effector	HB853_09795	Listeria welshimeri	88
A0AFZ6	Uncharacterized protein	lwe0510	Listeria welshimeri serovar 6b ATCC 35897	88
C5NV31	Uncharacterized protein	GEMHA0001_1408	Gemella haemolysans ATCC 10379	97

A0A2L0D3S0	TIGR04197 family type VII secretion effector	C0J00_04045	Streptococcus pluranimalium	119
A0A2X3XH13	Type VII secretion effector	NCTC11085_01347	Streptococcus sanguinis	97
F3UAG5	Uncharacterized protein	HMPREF9393_0458	Streptococcus sanguinis SK1056	97
A0A7H8UYP6	TIGR04197 family type VII secretion effector	FDP16_01520	Streptococcus sanguinis	90
A0A427Z4K1	Uncharacterized protein	D8889_08520	Streptococcus sanguinis	90
A0A0J6L9L6	Type VII secretion effector	VK90_07905	Bacillus sp. LK2	99
F0IN34	Uncharacterized protein	HMPREF9383_1537	Streptococcus sanguinis SK150	104
C5NV27	Uncharacterized protein	GEMHA0001_1404	Gemella haemolysans ATCC 10379	97
A0A6I3IT94	TIGR04197 family type VII secretion effector	GGH90_02870	Streptococcus sp. zg-36	86
A0A6I3I620	TIGR04197 family type VII secretion effector	GGG87_02865	Streptococcus sp. zg-86	86
A0A6I4RB72	TIGR04197 family type VII secretion effector	GGH11_02895	Streptococcus sp. zg-70	102
A0A1E5GIS0	Type VII secretion effector	BCR25_08220	Enterococcus termitis	96
W7C2R9	Uncharacterized protein	MFLO_13765	Listeria floridensis FSL S10-1187	96
A0A2C6WMQ9	TIGR04197 family type VII secretion effector	BTJ66_11860	Staphylococcus edaphicus	91

A0A5A7ZNX9	TIGR04197 family type VII secretion effector	FKX92_06255	Streptococcus sanguinis	90
A0A1E5GX99	Type VII secretion effector	BCR23_04625	Enterococcus quebecensis	96
A0A2X3V3S3	Type VII secretion effector	D8883_04730 NCTC11085_00302	Streptococcus sanguinis	90
A0A2N6SD26	TIGR04197 family type VII secretion effector	CJ218_07575	Gemella sanguinis	94
A0A1E5H6D9	Uncharacterized protein	BCR24_09880	Enterococcus ureilyticus	92
A0A7Z7QU85	Type VII secretion effector	NCTC8183_01311	Streptococcus agalactiae	126
F3UDH1	Uncharacterized protein	HMPREF9393_1578	Streptococcus sanguinis SK1056	90
J4X2D6	Type VII secretion effector, TIGR04197 family	HMPREF1150_0118	Streptococcus sp. AS14	90
A0A0F5MK39	Uncharacterized protein	RN86_02680	Streptococcus gordonii	116
A0A428AH08	Uncharacterized protein	D8875_04305	Streptococcus sanguinis	90
A0A2I1Z9Q6	TIGR04197 family type VII secretion effector	CYK23_08645	Streptococcus salivarius	90
A0A841YI15	TIGR04197 family type VII secretion effector	HB844_13830	Listeria fleischmannii	97
A0A2N6SD59	TIGR04197 family type VII secretion effector	CJ218_07595	Gemella sanguinis	94
A0A2V3VWP8	Type VII secretion	DFR56_108171	Pseudogracilibacillus auburnensis	88

	effector (TIGR04197 family)			
A0A841YHX4	TIGR04197 family type VII secretion effector	HB844_13140	Listeria fleischmannii	96
A0A7X1CAH5	TIGR04197 family type VII secretion effector	HCJ38_14380	Listeria immobilis	97
A0A1J4HAR3	Type VII secretion protein	HMPREF3241_05535	Staphylococcus sp. HMSC34G04	91
A0A3D8TTD4	Uncharacterized protein	UR08_00425	Listeria kieliensis	97
F0FPX7	Uncharacterized protein	HMPREF9392_0404	Streptococcus sanguinis SK678	90
F2CGJ8	Uncharacterized protein	HMPREF9391_1993	Streptococcus sanguinis SK408	90
F0ISH9	Uncharacterized protein	HMPREF9384_0791	Streptococcus sanguinis SK160	90
G5JNA7	Uncharacterized protein	STRCR_0144	Streptococcus criceti HS-6	93
A0A1E5GH45	Uncharacterized protein	BCR21_07315	Enterococcus ureasiticus	92
A0A7I0BHX0	TIGR04197 family type VII secretion effector	E1N03_11860	Staphylococcus epidermidis	91
A0A829M3W1	Type VII secretion protein	M453_0212855	Staphylococcus epidermidis CIM40	91
R2SNU8	Type VII secretion effector	UAY_02591	Enterococcus moraviensis ATCC BAA-383	96
A0A0B7GN05	Putative type VII secretion effector	SSV_1921	Streptococcus sanguinis	90
W7B2A2	Uncharacterized protein (Fragment)	MAQA_04586	Listeria aquatica FSL S10-1188	82
A0A1H9PSA2	Type VII secretion effector,	SAMN04488559_10172	Isobaculum melis	94

	SACOL2603 family			
W7B6K0	Uncharacterized protein (Fragment)	MAQA_04296	Listeria aquatica FSL S10-1188	83
A0A841YE47	TIGR04197 family type VII secretion effector	HB844_07260	Listeria fleischmannii	90
A0AK27	Uncharacterized protein	lwe1941	Listeria welshimeri serovar 6b ATCC 35897	97
A0A242AUE1	Uncharacterized protein	A5821_000621	Enterococcus sp. 7F3_DIV0205	92
A0A7X0Y3T3	TIGR04197 family type VII secretion effector	HCA69_08900	Listeria grandensis	90
A0A7X1C884	TIGR04197 family type VII secretion effector	HCJ38_03345	Listeria immobilis	97
W7B9C0	Uncharacterized protein	MAQA_15976	Listeria aquatica FSL S10-1188	97
A0A172Q5Q7	Uncharacterized protein	A0O21_01495	Streptococcus pantholopis	90
A0A7X0XCD6	TIGR04197 family type VII secretion effector	HCI99_06655	Listeria booriae	90
A0A239X809	Type VII secretion effector	SAMEA4504048_01597	Streptococcus acidominimus	105
V6Z4V5	Uncharacterized protein	SAG0136_11275	Streptococcus agalactiae LMG 14747	105
A0A540UVH0	TIGR04197 family type VII secretion effector	FH692_06345	Streptococcus suis	106
W7CDF0	Uncharacterized protein	MFLO_01075	Listeria floridensis FSL S10-1187	97
A0AKF5	Uncharacterized protein	lwe2069	Listeria welshimeri serovar 6b ATCC 35897	97

A0A7I0AJ13	TIGR04197 family type VII secretion effector	E1N03_09545	Staphylococcus epidermidis	91
A0A7X1C121	TIGR04197 family type VII secretion effector	HB856_09015	Listeria booriae	96
A0A2K4FCE9	TIGR04197 family type VII secretion effector	CD039_08645	Staphylococcus argensis	91
Q8DZR6	Uncharacterized protein	SAG1032	Streptococcus agalactiae serotype V ATCC BAA-611	85
A0A1F0CEK0	Uncharacterized protein	HMPREF2570_04395	Streptococcus sp. HMSC069D09	85
J8J5K7	Uncharacterized protein	IIO_06123	Bacillus cereus VD115	91
A0A1E5L0N0	Uncharacterized protein	BCR26_07815	Enterococcus rivorum	103
C0MDX1	Uncharacterized protein	SZO_07980	Streptococcus equi subsp. zooepidemicus (strain H70)	104
A0A076Z409	TIGR04197 family type VII secretion effector (Type VII secretion effector)	C4618_05905 D5F95_10620 DK41_05465 NCTC6175_01412 NCTC8185_02368	Streptococcus agalactiae	116
A0A829IEV4	Uncharacterized protein	SAG0014_09640	Streptococcus agalactiae FSL S3-586	116
Q8E5G5	Uncharacterized protein	gbs1067	Streptococcus agalactiae serotype III (strain NEM316)	116
A0A243G320	Type VII secretion effector	BK774_26435	Bacillus thuringiensis	91
A0A428IGV6	Uncharacterized protein	D8844_06495	Streptococcus oralis	121
A0A2S7RWC9	TIGR04197 family type VII secretion effector	CUS89_04340	Enterococcus mundtii	93

Table S2.3. Accession codes and sequence information for LapD1 homologs identified with one iteration of JackHMMER.

Entry	Protein names	Gene names	Organism	Length
A0A1F0ZSZ0	Type VII secretion protein	HMPREF2917_09355	Streptococcus sp. HMSC061E03	117
A0A359YHE7	Uncharacterized protein	SPADD19_01412	Streptococcus parasanguinis	117
I1ZK44	Uncharacterized protein	Spaf_0401	Streptococcus parasanguinis FW213	117
A0A2I1TT29	TIGR04197 family type VII secretion effector	CYK20_05490	Streptococcus parasanguinis	117
A0A6I3PAZ6	TIGR04197 family type VII secretion effector	GMC80_04760 GMC84_06705	Streptococcus parasanguinis	117
A0A1V0H196	TIGR04197 family type VII secretion effector	A6J85_03505	Streptococcus gordonii	118
A0A0F5MM43	Type VII secretion protein	RN86_02705	Streptococcus gordonii	118
S7XKY2	Type VII secretion protein	M059_05530	Streptococcus mitis 18/56	118
A0A1X1L326	Type VII secretion effector	B7692_08470 B7696_07565 B7700_09665	Streptococcus mitis	118
A0A178KGQ9	Type VII secretion protein	A3Q39_01930	Streptococcus sp. CCUG 49591	118
A0A414PGR1	TIGR04197 family type VII secretion effector	DW666_08555	Streptococcus parasanguinis	117
F8DHG1	Uncharacterized protein	HMPREF0833_11761	Streptococcus parasanguinis ATCC 15912	117
A0A3R9LZL4	Uncharacterized protein	D8803_08265	Streptococcus oralis	119
A0A428EFW5	Uncharacterized protein	D8839_01320	Streptococcus mitis	119
E6KIQ0	Uncharacterized protein	HMPREF8578_0115	Streptococcus oralis ATCC 49296	119
A0A8B1YMV5	TIGR04197 family type VII secretion effector	J4854_01600	Streptococcus lactarius	117
F9LWN2	Uncharacterized protein	HMPREF9965_0735	Streptococcus mitis bv. 2 str. SK95	119
A0A7H9FG17	TIGR04197 family type VII secretion effector	HRE59_00320	Streptococcus oralis subsp. oralis	119
A0A3R9PR96	Uncharacterized protein	D8860_09790	Streptococcus oralis	119

A0A1X1IPJ3	Type VII secretion effector	B7710_00065	Streptococcus oralis subsp. oralis	119
A0A139PJZ1	Uncharacterized protein	SORDD21_01112	Streptococcus oralis	119
A0A3L8GDQ6	TIGR04197 family type VII secretion effector	DIY07_08815	Streptococcus iniae (Streptococcus shiloi)	116
A0A178KI70	Type VII secretion protein	A3Q39_01960	Streptococcus sp. CCUG 49591	121
A0A1B1IDA9	Type VII secretion protein	AXF18_01820	Streptococcus sp. oral taxon 064	117
A0A427ZT45	Uncharacterized protein	D8882_08140	Streptococcus sanguinis	128
A3CR33	Uncharacterized protein	SSA_2276	Streptococcus sanguinis SK36	128
A0A3R9JBV3	Uncharacterized protein	D8860_05090	Streptococcus oralis	117
K0ZUT2	Uncharacterized protein	GMD4S_06157	Streptococcus sp. GMD4S	117
A0A3R9FWZ7	Uncharacterized protein	D8894_04895	Streptococcus oralis	117
K1A200	Uncharacterized protein	GMD6S_07863	Streptococcus sp. GMD6S	117
E6KIR3	Uncharacterized protein	HMPREF8578_0128	Streptococcus oralis ATCC 49296	117
A0A1X1IMY5	Type VII secretion effector	B7710_01125	Streptococcus oralis subsp. oralis	117
I0Q2A4	Type VII secretion effector, TIGR04197 family	HMPREF1115_1417	Streptococcus oralis SK610	117
F3UNP7	Uncharacterized protein	HMPREF9389_0455	Streptococcus sanguinis SK355	128
A0A1X1HVT4	Type VII secretion effector	B7714_09150	Streptococcus oralis subsp. oralis	117
S7XHE0	Type VII secretion protein	M059_05500	Streptococcus mitis 18/56	121
A0A1X1KD41	Type VII secretion effector	B7692_08440 B7696_07595	Streptococcus mitis	121
A0A428IGV6	Uncharacterized protein	D8844_06495	Streptococcus oralis	121
E3CF41	Uncharacterized protein	HMPREF9626_1803	Streptococcus parasanguinis F0405	117
A0A1X1JX30	Type VII secretion effector	B7700_09695	Streptococcus mitis	121
F0FHF7	Uncharacterized protein	HMPREF9388_2140	Streptococcus sanguinis SK353	128
A0A1X1J482	Type VII secretion effector	B7708_00965	Streptococcus oralis subsp. dentisani	121

A0A3R9KBB5	Uncharacterized protein	D8801_04895	Streptococcus oralis	121
A0A076Z409	TIGR04197 family type VII secretion effector (Type VII secretion effector)	C4618_05905 D5F95_10620 DK41_05465 NCTC6175_01412 NCTC8185_02368	Streptococcus agalactiae	116
Q8E5G5	Uncharacterized protein	gbs1067	Streptococcus agalactiae serotype III strain NEM316	116
A0A829IEV4	Uncharacterized protein	SAG0014_09640	Streptococcus agalactiae FSL S3-586	116
A0A0E1EMX7	TIGR04197 family type VII secretion effector	AX245_04155 C4618_11685 C6N07_05895 RDF_1030	Streptococcus agalactiae	111
A0A837KW31	Uncharacterized protein	WA04_10840	Streptococcus agalactiae	116
A0A4R5G605	TIGR04197 family type VII secretion effector	E0E04_02150	Streptococcus vicugnae	118
A0A7D4GGP0	TIGR04197 family type VII secretion effector	FOC63_06865	Streptococcus gallolyticus	118
A0A139N5A4	Uncharacterized protein	SCRDD08_00137	Streptococcus cristatus	121
A0A4T2GKS9	TIGR04197 family type VII secretion effector	FAJ39_07705	Streptococcus suis	109
A0A7X2UFL6	Type VII secretion effector	NCTC3858_01464	Streptococcus uberis	111
A0A7Z0VGH5	Uncharacterized protein	TH70_0120	Streptococcus agalactiae	111
A0A8B4IN87	Type VII secretion effector	NCTC3858_00392	Streptococcus uberis	108
A0A7Z7QU85	Type VII secretion effector	NCTC8183_01311	Streptococcus agalactiae	126
Q8DZR6	Uncharacterized protein	SAG1032	Streptococcus agalactiae serotype V ATCC BAA-611	85
A0A1F0CEK0	Uncharacterized protein	HMPREF2570_04395	Streptococcus sp. HMSC069D09	85
A0A1E5KUF8	Uncharacterized protein	BCR26_04480	Enterococcus rivorum	93
A0A3R9NTY4	Uncharacterized protein	D8879_08845	Streptococcus sanguinis	90
A0A1E5KVA8	Uncharacterized protein	BCR26_15430	Enterococcus rivorum	93
A0A0F5MK39	Uncharacterized protein	RN86_02680	Streptococcus gordonii	116

A0A7H8V643	TIGR04197 family type VII secretion effector	FFV08_03635	Streptococcus sanguinis	125
F3USM5	Uncharacterized protein	HMPREF9389_1833	Streptococcus sanguinis SK355	90
A0A3R9IAM7	TIGR04197 family type VII secretion effector	D8887_08455 FFV08_05580	Streptococcus sanguinis	90
A0A427ZP46	Uncharacterized protein	D8886_07895	Streptococcus sanguinis	90
A0A5A7ZT92	TIGR04197 family type VII secretion effector	FKX92_00595	Streptococcus sanguinis	124
A0A139NQ79	Uncharacterized protein	STRDD11_02464	Streptococcus sp. DD11	89
A0A540UNN4	TIGR04197 family type VII secretion effector	FH692_10960	Streptococcus suis	134
A0A0Z8X7W8	Type VII secretion effector	ERS132372_01527 ERS132399_02390	Streptococcus suis	111
A0A116LSC7	TIGR04197 family type VII secretion effector (Type VII secretion effector)	ERS132406_02093 ERS132410_02193 FAJ36_02915	Streptococcus suis	108
A0A4P7WT47	TIGR04197 family type VII secretion effector	E8M06_09960	Streptococcus suis	108
A0A0Z8DGM0	TIGR04197 family type VII secretion effector (Type VII secretion effector)	E8M06_09990 ERS132392_00702 JZY07_10375	Streptococcus suis	108
A0A0S3K715	Type VII secretion effector	ATZ33_01365	Enterococcus silesiacus	104
A0A1E5HGI4	Type VII secretion effector	BCR24_01530	Enterococcus ureilyticus	104
A0A0B7GN05	Putative type VII secretion effector	SSV_1921	Streptococcus sanguinis	90
A0A239X809	Type VII secretion effector	SAMEA4504048_01597	Streptococcus acidominimus	105
V6Z4V5	Uncharacterized protein	SAG0136_11275	Streptococcus agalactiae LMG 14747	105
A0A540UVH0	TIGR04197 family type VII secretion effector	FH692_06345	Streptococcus suis	106
A0A242AX92	Uncharacterized protein	A5821_001500	Enterococcus sp. 7F3_DIV0205	99

A0A139QYV5	Uncharacterized protein	SGADD02_00817 SGADD03_01202	Streptococcus gallolyticus	90
A0A380K862	Type VII secretion effector	NCTC13767_01892	Streptococcus gallolyticus	90
A0A1E5GK70	Type VII secretion effector	BCR25_06445	Enterococcus termitis	104
F0IN34	Uncharacterized protein	HMPREF9383_1537	Streptococcus sanguinis SK150	104
A0A2G3NUY4	TIGR04197 family type VII secretion effector	CS009_05415 CS010_03220	Streptococcus macedonicus	90
A0A3R9G5C1	Uncharacterized protein	D8887_07710	Streptococcus sanguinis	92
A0A0A0DFU7	Uncharacterized protein	SSIN_0557	Streptococcus sinensis	104
A0A2L0D3S0	TIGR04197 family type VII secretion effector	C0J00_04045	Streptococcus pluranimalium	119
A0A4T2H474	TIGR04197 family type VII secretion effector	FAJ36_02885	Streptococcus suis	134
F5X0A7	Uncharacterized protein	SGGB_0839	Streptococcus gallolyticus ATCC 43143	90
A0A7D4GS34	TIGR04197 family type VII secretion effector	FOC63_08560	Streptococcus gallolyticus	90
A0A1S5WBI2	Uncharacterized protein	BTR42_04595	Streptococcus gallolyticus subsp. gallolyticus DSM 16831	90
A0A359YGK2	Uncharacterized protein	SPADD19_01110	Streptococcus parasanguinis	89
A0A242H2M5	Uncharacterized protein	A5866_002123	Enterococcus sp. 12C11_DIV0727	103
A0A7H8UYP6	TIGR04197 family type VII secretion effector	FDP16_01520	Streptococcus sanguinis	90
A0A0U2NRL1	Type VII secretion effector	ATZ35_10775	Enterococcus rotai	103
A0A4Q2FH31	TIGR04197 family type VII secretion effector	DF218_03565	Streptococcus parasanguinis	89
A0A1I7FJ84	Type VII secretion effector, SACOL2603 family	SAMN05660328_101420	Streptococcus gallolyticus	90
A0A1E5GE80	Type VII secretion effector	BCR21_11960	Enterococcus ureasiticus	103

A0A428AH08	Uncharacterized protein	D8875_04305	Streptococcus sanguinis	90
A0A2I1Z9Q6	TIGR04197 family type VII secretion effector	CYK23_08645	Streptococcus salivarius	90
F3UDH1	Uncharacterized protein	HMPREF9393_1578	Streptococcus sanguinis SK1056	90
I2NMG3	Uncharacterized protein	HMPREF9971_1232	Streptococcus parasanguinis F0449	113
A0A242LA78	Uncharacterized protein	A5881_003608	Enterococcus termitis	104
A0A6I3PR01	TIGR04197 family type VII secretion effector	GMC95_02245	Streptococcus parasanguinis	94
A0A0E2IQB7	Uncharacterized protein	HMPREF1654_01870	Streptococcus intermedius ATCC 27335	91
R2QN21	Type VII secretion effector	UAY_02986	Enterococcus moraviensis ATCC BAA-383	98
A0A139MU55	Uncharacterized protein	STRDD04_00268	Streptococcus sp. DD04	97
A0A6N3CT23	Uncharacterized protein	SPLFYP13_01158	Streptococcus parasanguinis	89
A0A1F1A3X5	Uncharacterized protein	HMPREF2917_04405	Streptococcus sp. HMSC061E03	89
A0A427Z4K1	Uncharacterized protein	D8889_08520	Streptococcus sanguinis	90
A0A1F0AWW4	Uncharacterized protein	HMPREF2686_08175	Streptococcus sp. HMSC057G03	89
J4X2D6	Type VII secretion effector, TIGR04197 family	HMPREF1150_0118	Streptococcus sp. AS14	90
A0A0B7GL02	Putative type VII secretion effector	SSV_1220	Streptococcus sanguinis	97
A0A4Q5BT34	TIGR04197 family type VII secretion effector	GMC84_09185 GMC94_02205	Streptococcus parasanguinis	89
F8DGG8	Uncharacterized protein	HMPREF0833_10386	Streptococcus parasanguinis ATCC 15912	94
E8K4F1	Uncharacterized protein	HMPREF8577_0436	Streptococcus parasanguinis ATCC 903	99
